# Seasonal Dietary Shifts Alter the Gut Microbiota of Avivorous Bats: Implication for Adaptation to Energy Harvest and Nutritional Utilization

**DOI:** 10.1128/mSphere.00467-21

**Published:** 2021-08-04

**Authors:** Lixin Gong, Boyu Liu, Hui Wu, Jiang Feng, Tinglei Jiang

**Affiliations:** a Jilin Provincial Key Laboratory of Animal Resource Conservation and Utilization, Northeast Normal University, Changchun, China; b Key Laboratory of Vegetation Ecology of Education Ministry, Institute of Grassland Science, Northeast Normal University, Changchun, China; c College of Animal Science and Technology, College of Veterinary Medicine, Jilin Agricultural University, Changchun, China; d College of Life Science, Jilin Agricultural University, Changchun, China; University of Wisconsin-Madison

**Keywords:** bats, dietary niche expansion, diet-host-microbiota-physiology relationship, energy and nutritional requirements

## Abstract

Plasticity in the microbial community composition and function can permit the host to adapt to ecological, environmental, and physiological changes. Much of the information on the gut microbiota-host relationship to date derives from studies of laboratory model organisms, while little is known concerning wild animals and their ecological relevance to gut microbes. It is also unclear how microbial community composition and activity adapt to changes in diet and energy, nutritional requirements, and utilization induced by dietary expansion from invertebrates to vertebrates. The great evening bat (Ia io) is both an insectivore and an avivore (that is, a bird-eater), and thus provides an opportunity to investigate the diet-host-microbiota-physiology relationship. Here, we investigated this relationship by using 16S rRNA amplicon sequencing and functional prediction in adult males of *I. io*. We found that gut microbial diversity was similar, while microbial community structures were significantly different between insectivorous and avivorous diets. Moreover, increases in the relative abundance of *Firmicutes* and the *Firmicutes*-to-*Bacteroidetes* ratio, changes in carbohydrate and nucleotide metabolism, and a decrease in Pseudomonas were associated with higher energy demands for hunting birds and with fat storage for entering hibernation and migration. These findings demonstrated that seasonal dietary shifts drive a significant change in the composition and function of gut microbiomes, thereby facilitating adaptation to the challenging avian dietary niche in bats. These results suggest that the gut microbial communities can constantly respond to alterations in diets, potentially facilitating the diversity of wild animal dietary niches, and enhance our understanding of the diet-host-microbiota-physiology relationship.

**IMPORTANCE** The coevolution between the host and its gut microbes can promote an animal’s adaptation to its specific ecological niche and changes in energy and nutritional requirements. This study focused on an avivorous bat, the great evening bat (*Ia io*), to investigate how seasonal dietary shifts affect the gut microbial composition and function, thereby facilitating adaptation to an avian diet. We found that seasonal dietary shifts driving a significant change in the composition and function of gut microbiomes in *I. io* were associated with higher energy demands for hunting birds and fat storage for entering hibernation and migration. Our study provides novel insight into the role of gut bacteria in generating ecological diversity and flexibility in wild mammals. The results are valuable for clarifying the complicated host-microbiota-physiology relationship in a dietary niche expansion context.

## INTRODUCTION

Studying animal microbiomes can help us answer pivotal questions related to host evolution and ecology (1, 2). Mounting studies have demonstrated that gut microbial communities play an essential role in driving host nutrition, energetics, health, behavior, and thus fitness, as the microbiome provides the host flexibility in its capacity to handle ecological and environmental changes (3–7). In turn, host diet, genetics, age, sex, behavior, and external environmental factors all induce variation in the gut microbiota (8–12). However, much of the information on the gut microbiota-host relationship to date is from studies of laboratory model organisms. Thus, little is known concerning wild animals and their ecological relevance to gut microbes (7).

Dietary niche expansion is an important process for the adaptation of animals to seasonal and environmental changes (13). Diet changes represent physiological and behavioral challenges for the host, often in association with different energy and nutritional requirements. Diet is a key factor that shapes the composition and function of gut microbiota in animals as well as in humans (14–16). It would be expected that gut microbiota likely provides essential functions related to specialized diets, including the host’s food assimilation efficiency, metabolic rate, energy harvest, and nutritional utilization, and thus allow hosts to expand their niches (7). For example, in spite of the low digestibility of the cellulose and hemicellulose in the unique bamboo diet of the giant panda (Ailuropoda melanoleuca), its gut microbiome contains genes related to cellulose degradation in enzymes that contribute to raw fiber degradation and nutrient utilization, facilitating adaptation to the unique bamboo diet (17, 18). Moreover, variation in energy and nutritional requirements for hunting different food resources may lead to the changes in gut microbiota (7). Although the relationship between host diet and the gut microbiota has been extensively investigated in many contexts, universality is still limited (7). Thus, attempts to clarify the diet-host-microbiota-physiology relationship remain a challenge in dietary niche expansion.

Bats are an excellent study system for investigating questions concerning the role of microbes in shaping host physiology, evolution, and fitness, due to their taxonomic, ecological, and dietary diversity (19, 20). Additionally, bats are currently an unexploited resource for understanding microbiome evolution in mammals (20). Predation of bats on birds (namely, avivorous bats) is a rare process in the natural world. Of 1,400 bat species (21), only three temperate-subtropical species, Ia io, Nyctalus lasiopterus, and Nyctalus aviator, are known to prey on insects in summer but also known to hunt nocturnally migrating birds in spring and autumn (22–24). Thus, avivory in bats represents a case of dietary niche expansion from low- to high-quality food resources, since birds have higher nutritional value than invertebrate insects (25). However, it is unclear how avivorous bats adjust their gut microbiota to adapt to changes in diet and energy and nutritional requirements induced by the dietary shift toward birds. Moreover, it may be due to the different microbiota transiently brought in with the consumed bird prey. The great evening bat *I. io* is the only known avivorous bat in southern China. These bats feed on different prey species according to seasonal variation in food resources. They mainly forage on small passerines during bird migrations in spring and autumn, and they mostly feed on insects in summer (24, 26). Moreover, our previous study demonstrated by high-throughput sequencing that *I. io* preys on at least 22 species of passerine birds (24). This dietary shift provides an opportunity for examining the composition and function of gut microbiota during the dietary expansion from an insectivorous diet to a carnivorous (avivorous) diet.

In this study, we investigated the diet-host-microbiota-physiology relationship via seasonal dietary shifts (insects in summer versus birds in autumn) in adult males of *I. io* by using 16S rRNA amplicon sequencing, advanced amplicon sequence variant (ASV) analysis, and PICRUSt2 to predict functional profiles. We tested the following two hypotheses. First, since plasticity in the microbial community composition can permit the host to adapt to changing food resources (27), we hypothesized that seasonal shifts in diet are associated with changes in the composition of gut microbiota in *I. io*. Second, predation by bats on birds is associated with higher energy demands than predation on insects, because birds are much larger in body size and fly faster than insects (28). Moreover, enough fat storage in autumn before entering hibernation and migration is pivotal for survival during the hibernation period and the journey of migration in bats (29, 30). Thus, we hypothesized that changes in the composition and function of gut microbiota in connection with the absorption and utilization of food energy and nutrition in *I. io* would be observed when the bats feed on birds in autumn and the changes would be beneficial to the bat host (i.e., increases in body mass for hibernation and migration).

## RESULTS

After quality processing, our sequencing effort obtained a total of 929,674 reads, an average of 29,052 ± standard deviation (SD) 6,289 sequences per sample (minimum 20,316; maximum 55,341; see Table S1 in the supplemental material), with 4,671 total ASVs. After the nonbacterial, chloroplast, and mitochondrial ASVs were excluded and rarefied, the remaining 4,522 ASVs were used for analysis. Additionally, there were no significant differences in forearm length (*Z* = −1.602, *P* = 0.109), while a significant difference in body mass (*Z* = −4.560, *P* < 0.001) of *I. io* was observed between insectivorous and avivorous diets (see Fig. 2A and Table S1).

10.1128/mSphere.00467-21.3 TABLE S1 Information on sampling, number of sequences per sample, and alpha diversity index of the gut microbiome of the great evening bat in this study. Download Table S1, DOCX file, 18 kb. Copyright © 2021 Gong et al. 2021 Gong et al. https://creativecommons.org/licenses/by/4.0/ This content is distributed under the terms of the Creative Commons Attribution 4.0 International license.

### Alpha and beta diversity.

**FIG 1:**
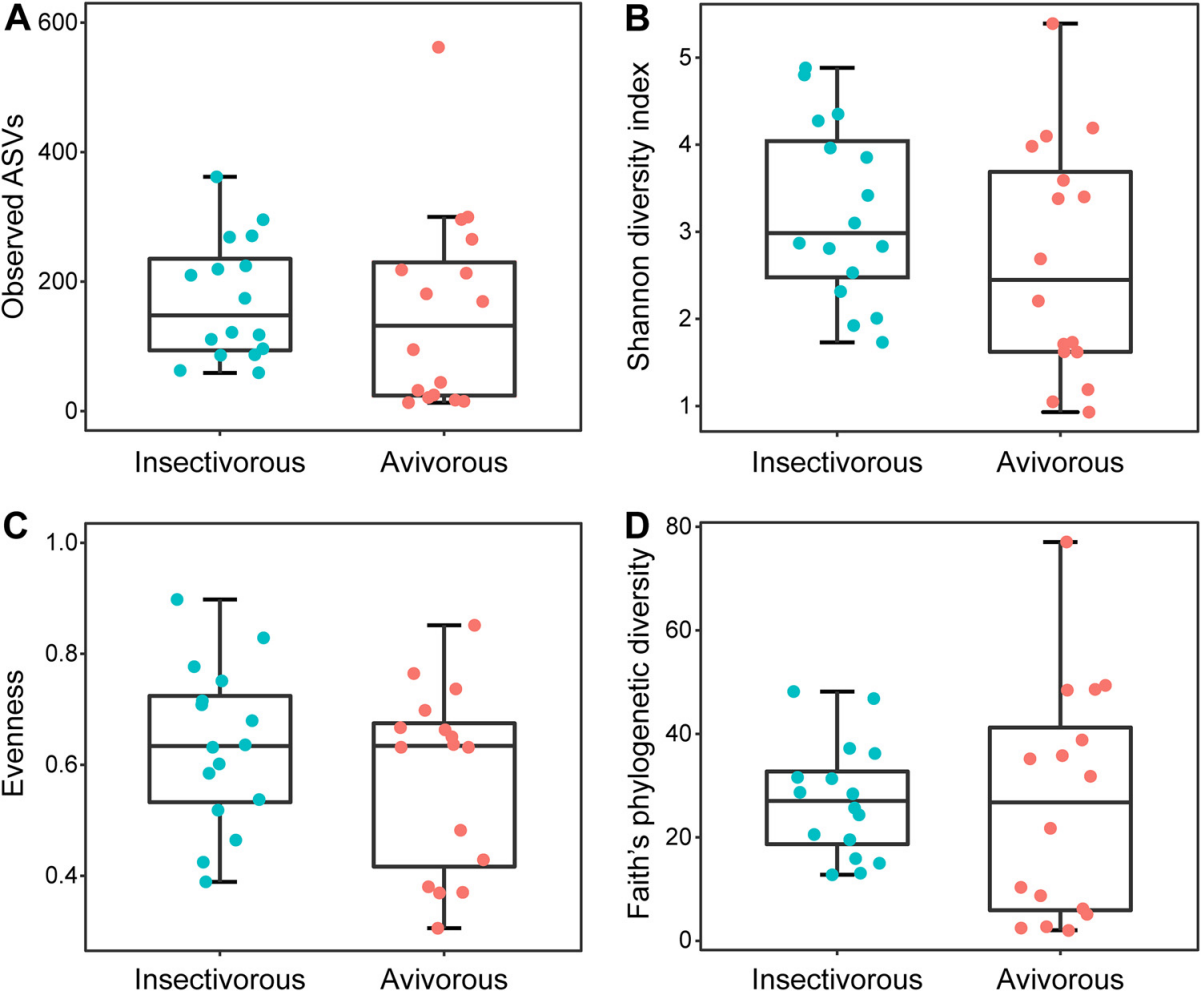
Alpha diversity indices of the gut microbiome community in the great evening bat between insectivorous and avivorous diets. (A) Observed ASVs. (B) Shannon diversity index. (C) Evenness. (D) Faith’s phylogenetic diversity. There were no significant differences in any of the alpha diversity indices; all *P* values were >0.05 (Table 1 and Table S1).

**FIG 2:**
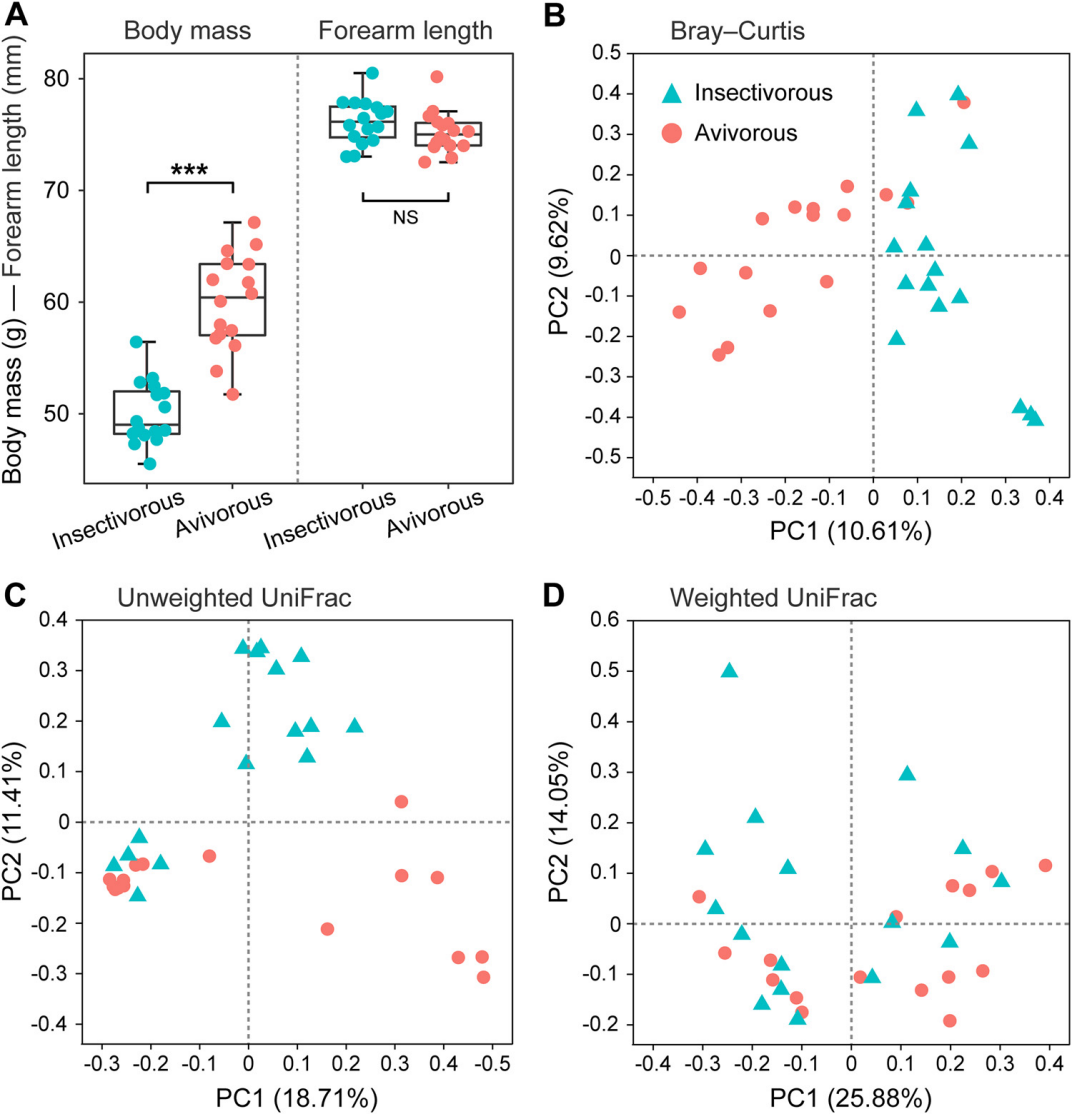
(A) Differences in body mass and forearm length of great evening bats between insectivorous and avivorous diets. ***, *P* < 0.001; NS, not significant. (B to D) Principal-coordinate analysis (PCoA) plots of great evening bat gut microbiome community structure between insectivorous and avivorous diets. (B) PCoA plot based on Bray-Curtis distance. (C) PCoA plot based on unweighted UniFrac distance. (D) PCoA plot based on weighted UniFrac distance.

There were no differences in the four metrics of gut microbial alpha diversity for *I. io* between insectivorous and avivorous diets (all *P* > 0.05; Fig. 1 and Table 1). Moreover, no effect of body mass on microbial diversity was observed (all *P* > 0.05; Table 2). Significant differences were observed in the beta diversity between insectivorous and avivorous diets (Fig. 2B to D). Principal-coordinate analysis (PCoA) of Bray-Curtis distance (permutational multivariate analysis of variance [PERMANOVA]: *R*^2^ = 0.0758, *P* = 0.001; Fig. 2B), unweighted UniFrac distance (PERMANOVA: *R*^2^ = 0.0743, *P* = 0.004; Fig. 2C), and weighted UniFrac distance (PERMANOVA: *R*^2^ = 0.0808, *P* = 0.014; Fig. 2D) matrices clearly showed gut microbial communities clustered by diet. Meanwhile, permutational analysis of multivariate dispersions (PERMDISP) showed that Bray-Curtis distance (*F *= 3.574, *P* = 0.078; Fig. S1A), unweighted UniFrac distance (*F *= 1.085, *P* = 0.318; Fig. S1B), and weighted UniFrac distance (*F *= 2.683, *P* = 0.099; Fig. S1C) were homogeneous dispersions.

**TABLE 1:** Differences in alpha diversity indices of the gut microbiome in great evening bats between insectivorous and avivorous diets

Alpha diversity index	Mean ± SD		*Z*	*P* value
	Insectivorous	Avivorous		
Observed ASVs	172.94 ± 93.53	154.13 ± 153.09	−1.112	0.266
Shannon diversity index	3.23 ± 1.02	2.67 ± 1.35	−1.489	0.136
Evenness	0.63 ± 0.15	0.58 ± 0.16	−0.848	0.396
Faith’s phylogenetic diversity	27.21 ± 11.06	26.53 ± 22.51	−0.302	0.763

**TABLE 2:** Simple linear regressions between body mass and microbial (alpha) diversity

Model: predictor—response	Estimate ± SE	*R* ^2^	*t*	*P* value
Body mass—Observed ASVs	0.002 ± 0.009	−0.032	0.196	0.846
Body mass—Shannon diversity index	−0.380 ± 0.927	−0.028	−0.410	0.685
Body mass—evenness	−2.741 ± 7.224	−0.028	−0.380	0.707
Body mass—Faith’s phylogenetic diversity	−0.023 ± 0.064	−0.029	0.355	0.725

10.1128/mSphere.00467-21.1 FIG S1 Permutational analysis of multivariate dispersions (PERMDISP) to test homogeneity of dispersions for Bray-Curtis distance (A), unweighted UniFrac distance (B), and weighted UniFrac distance (C). Left panel shows the distances to centroids on the first two PCoA axes for insectivorous and avivorous dietary groups; right panel shows a box plot of the distances to centroid for each dietary group. Download FIG S1, TIF file, 2,948 kb. Copyright © 2021 Gong et al. 2021 Gong et al. https://creativecommons.org/licenses/by/4.0/ This content is distributed under the terms of the Creative Commons Attribution 4.0 International license.

### Gut microbiota composition and changes.

**FIG 3:**
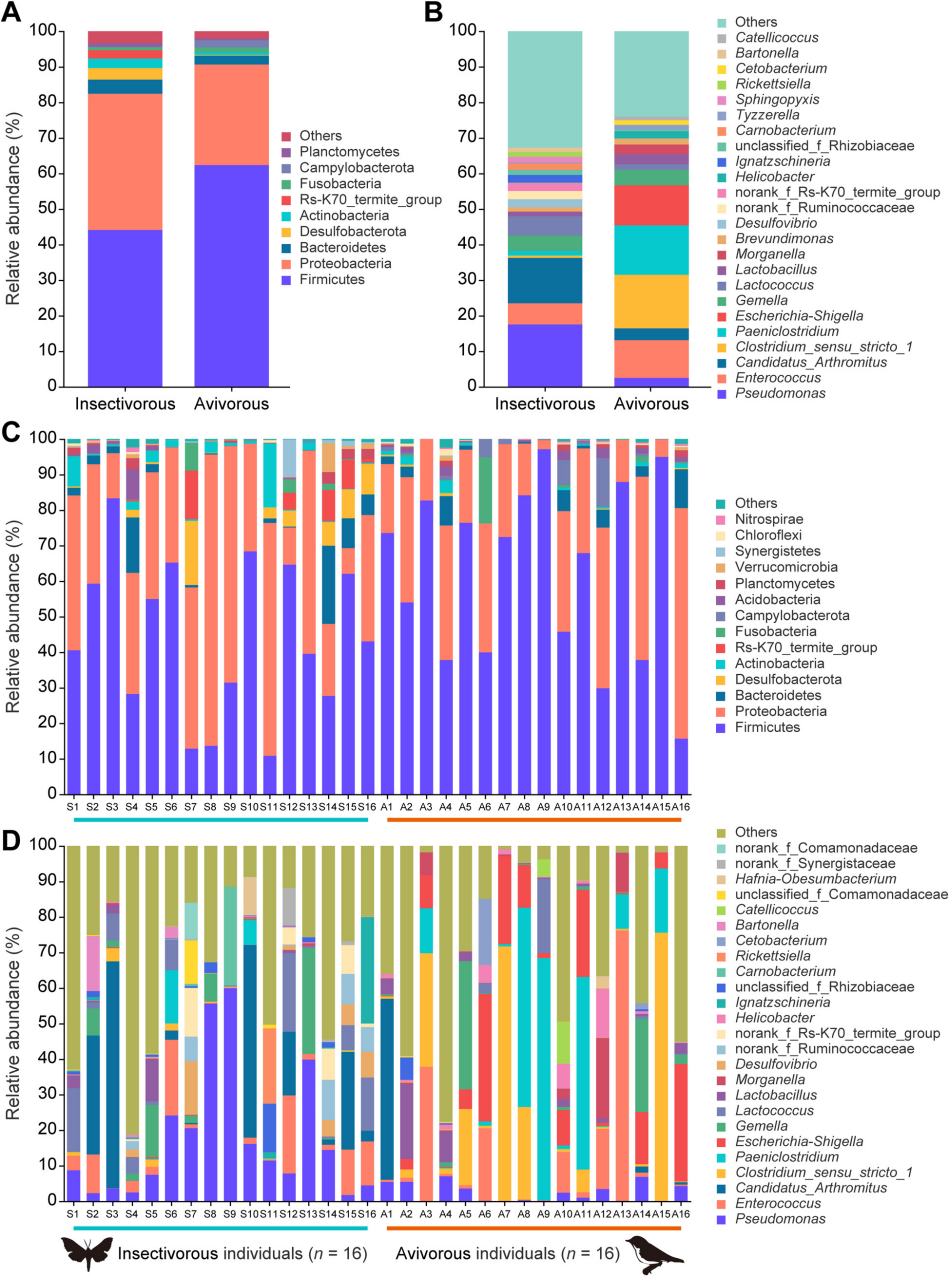
Composition of the gut microbiome community in great evening bats between insectivorous and avivorous diets. (A) The relative abundance of bacterial phyla across all samples. (B) The relative abundance of bacterial genera across all samples. (C) The relative abundance of bacterial phyla of each sample. (D) The relative abundance of bacterial genera of each sample. For panels C and D, each stacked bar represents an individual bat. In each panel, “Others” represents the sum of the relative abundances of all other phyla and genera.

**FIG 4:**
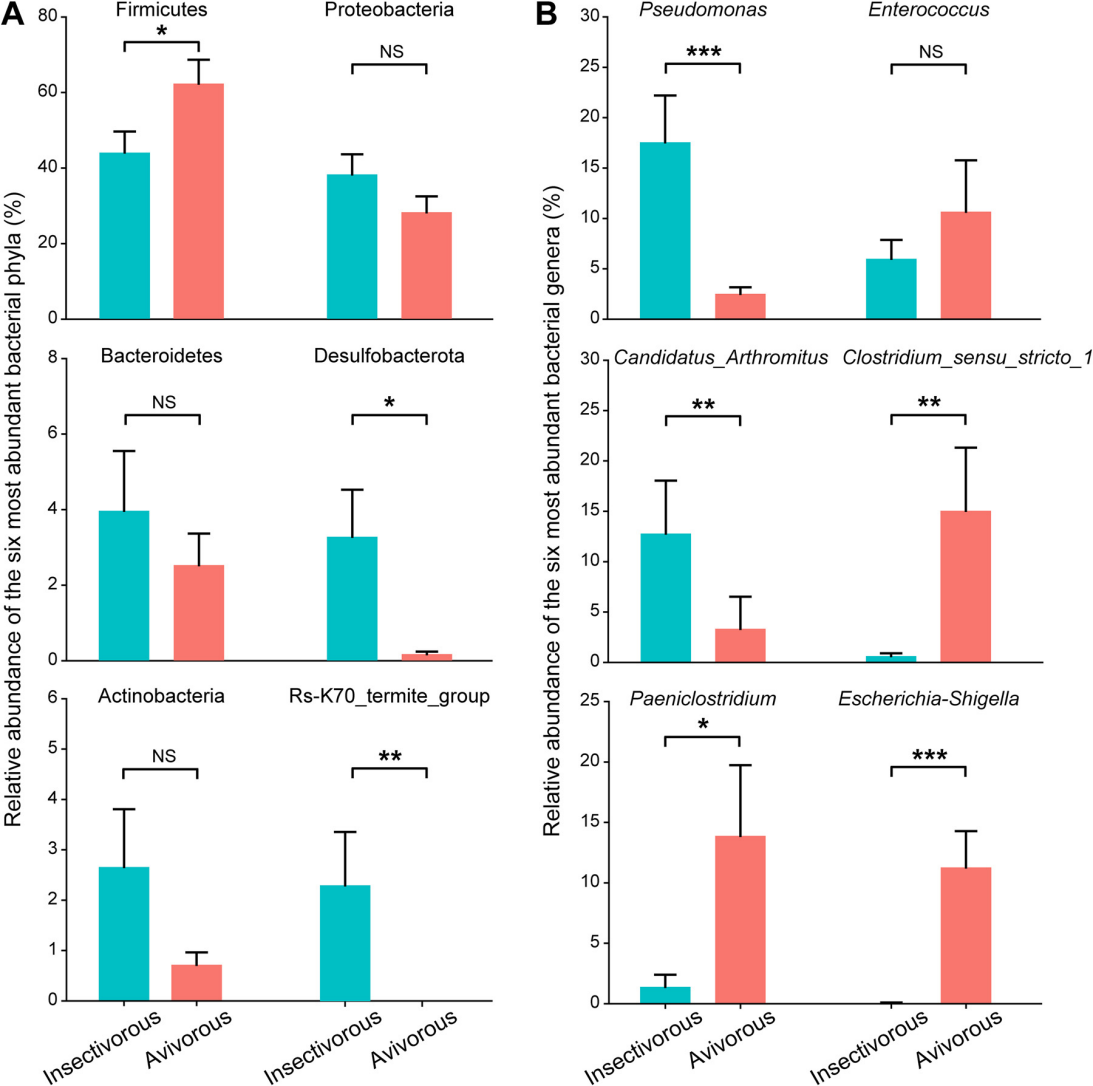
Comparison of gut microbial communities in great evening bats between insectivorous and avivorous diets at phylum-level and genus-level taxa. (A) Relative abundances of the six most abundant bacterial phyla. (B) Relative abundances of the six most abundant bacterial genera. Bars show mean ± standard error (SE). *, *P* < 0.05; **, *P* < 0.01; ***, *P* < 0.001; NS, not significant.

Taxonomic assignment clearly revealed that the dominant phyla were *Firmicutes* and *Proteobacteria*, which were present as core microbiome components across all individual bats (Fig. 3A and C). At the phylum level, the *Firmicutes* (relative abundance 44.1% in the insectivorous diet, 62.4% in the avivorous diet) and *Proteobacteria* (38.3%, 28.3%) dominated the gut microbiota, followed by *Bacteroidetes* (4.0%, 2.5%) between respective insectivorous and avivorous diets, respectively (Fig. 3A). Here, we compared relative abundances of the six most common bacterial phyla. *Firmicutes* increased significantly (*Z* = −2.073, *P* = 0.038), while *Desulfobacterota* (*Z* = −2.573, *P* = 0.010) and Rs-K70_termite_group (*Z* = −2.656, *P* = 0.008) decreased significantly in the avivorous diet compared to the insectivorous diet (Fig. 4A and Table S2). At the genus level, there were differences in the composition of the gut microbial community among individual bats in different diets (Fig. 3B and D). The relative abundance of gut microbiota in the insectivorous diet was dominated by Pseudomonas (17.6%) followed by “*Candidatus* Arthromitus” (12.8%) and *Enterococcus* (5.9%). However, the most abundant gut microbiota in the avivorous diet were mainly from the four genera *Clostridium_sensu_stricto_1* (15.1%), *Paeniclostridium* (13.9%), Escherichia-*Shigella* (11.3%), and *Enterococcus* (10.6%). The six most abundant bacterial genera were analyzed, and the results showed higher relative abundances of *Clostridium_sensu_stricto_1*, *Paeniclostridium*, and Escherichia-*Shigella*, while there were lower relative abundances of Pseudomonas and “*Candidatus* Arthromitus” in the avivorous diet compared with the insectivorous diet (all *P* < 0.05; Fig. 4B and Table S2). Altogether, we identified five phyla and 56 genera that displayed significant differences in relative abundance between diets (Table S3).

10.1128/mSphere.00467-21.4 TABLE S2 Relative abundances of the six most abundant bacterial phyla and genera of the gut microbial community in great evening bats between insectivorous and avivorous diets. Values shown are means ± SE. Significant results are in bold (*P* < 0.05). Download Table S2, DOCX file, 14 kb. Copyright © 2021 Gong et al. 2021 Gong et al. https://creativecommons.org/licenses/by/4.0/ This content is distributed under the terms of the Creative Commons Attribution 4.0 International license.

10.1128/mSphere.00467-21.5 TABLE S3 Significant differences in relative abundances at phylum and genus levels of the gut microbial community in great evening bats between insectivorous and avivorous diets. Values shown are means ± SD. Significance levels are *P* < 0.05. Download Table S3, DOCX file, 20 kb. Copyright © 2021 Gong et al. 2021 Gong et al. https://creativecommons.org/licenses/by/4.0/ This content is distributed under the terms of the Creative Commons Attribution 4.0 International license.

**FIG 5:**
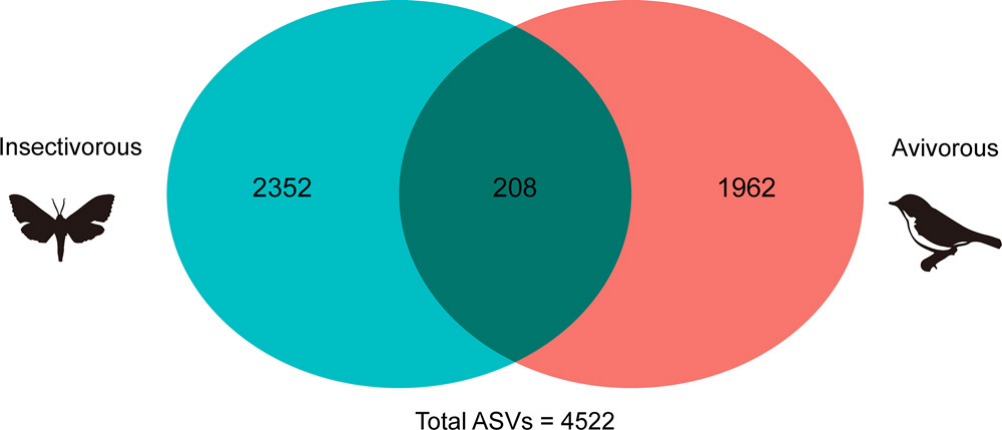
Venn diagram showing the overlapping number of ASVs between insectivorous and avivorous diets.

**FIG 6:**
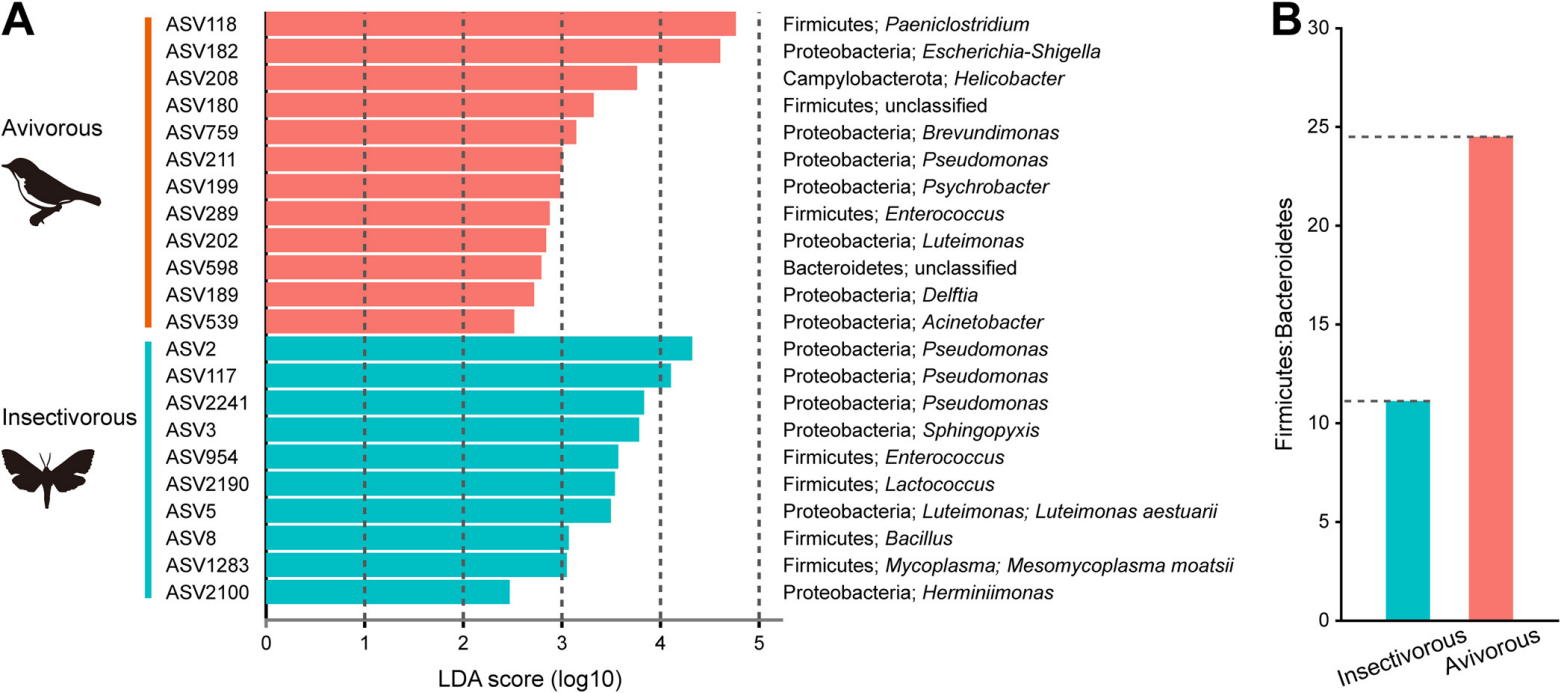
(A) The differentially represented ASVs and the LDA score between insectivorous and avivorous diets determined by LEfSe analysis. (B) The ratio of mean relative abundance of *Firmicutes* to mean relative abundance of *Bacteroidetes* in great evening bats between insectivorous and avivorous dietary groups.

We identified 2,560 ASVs in insectivorous diets and 2,170 ASVs in avivorous diets. These ASVs constituted a total of 4,522 ASVs, of which 208 were shared across the two diets (Fig. 5). Further, a linear discriminant analysis (LDA) effect size (LEfSe) analysis revealed that 22 ASVs differed significantly between the microbiome of insectivorous and avivorous diets (LDA score > 2, *P* < 0.05; Fig. 6A). Among these, seven ASVs belonged to the phylum *Firmicutes*; 13 ASVs belonged to the phylum *Proteobacteria*, and the other two belonged to the phyla *Bacteroidetes* and *Campylobacterota*. When considering an LDA score of >4 as having the most influence of ASVs on the difference between diets, we found that ASV118 belonged to the genus *Paeniclostridium* (phylum *Firmicutes*), and ASV182 belonged to the genus Escherichia-*Shigella* (phylum *Proteobacteria*) in the avivorous diet, with ASV2 and ASV117 belonging to the genus Pseudomonas (phylum *Proteobacteria*) in the insectivorous diet (Fig. 6A). In addition, we found that the mean relative abundance ratio of *Firmicutes* to *Bacteroidetes* was higher in the avivorous diet (24.65) than in the insectivorous diet (11.11) (Fig. 6B).

### Microbial function changes.

**FIG 7:**
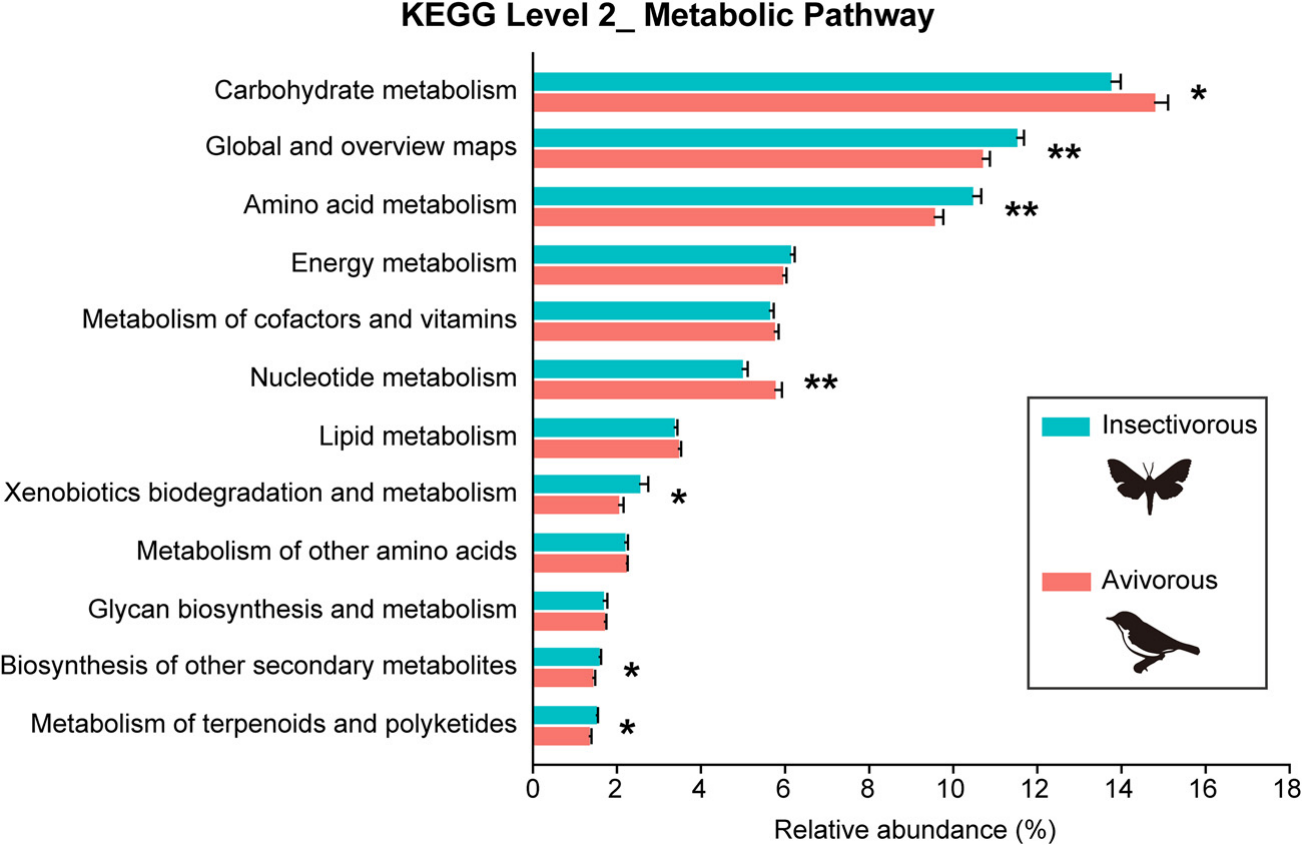
Predicted functions of microbial metagenomes based on the KEGG level 2 metabolism-related categories between insectivorous and avivorous diets. The relative abundance represents percentage of KEGG assignments in different metabolic categories. Bars show mean ± SE. *, *P* < 0.05; **, *P* < 0.01.

Our results showed that microbial functions clustered into 12 metabolic categories (Fig. 7). The relative abundance of seven predicted metabolism-related functional categories changed significantly between insectivorous and avivorous diets. Individual bats foraging on birds had a higher relative abundance of microbiota associated with carbohydrate metabolism (*Z* = −2.337, *P* = 0.019) and nucleotide metabolism (*Z* = −3.053, *P* = 0.002). However, individuals feeding on insects had higher relative abundance of microbiota related to global and overview maps (e.g., carbon and fatty acid metabolism; *Z* = −2.714, *P* = 0.007), amino acid metabolism (*Z* = −2.751, *P* = 0.006), xenobiotic biodegradation and metabolism (*Z* = −2.035, *P* = 0.042), biosynthesis of other secondary metabolites (*Z* = −2.261, *P* = 0.024), and metabolism of terpenoids and polyketides (*Z* = −2.563, *P* = 0.010) (Fig. 7 and Table S4).

10.1128/mSphere.00467-21.6 TABLE S4 PICRUSt2 showing predicted relative abundance of metabolism-related functional categories based on the second-level KEGG pathways. Values shown are means ± SE. Significant results are in bold (*P* < 0.05). Download Table S4, DOCX file, 14 kb. Copyright © 2021 Gong et al. 2021 Gong et al. https://creativecommons.org/licenses/by/4.0/ This content is distributed under the terms of the Creative Commons Attribution 4.0 International license.

## DISCUSSION

In this study, we found that seasonal dietary shifts were closely associated with changes in the beta diversity of gut microbiota in *I. io*, supporting our first hypothesis that diet changes are related to the composition of gut microbiota changes. Moreover, changes in the composition and function of gut microbes in individual bats with the avian diet, such as the increases in the relative abundance of *Firmicutes*, the *Firmicutes*-to-*Bacteroidetes* ratio, and carbohydrate and nucleotide metabolism along with a decrease in Pseudomonas, were associated with higher energy demands for hunting birds and with fat storage for entering hibernation and migration, supporting our second hypothesis that the composition and function of gut microbiota change in connection with the absorption and utilization of food energy and nutrition. These results demonstrated an indivisible relationship between the unique avian diet of *I. io*, its gut microbiota, and physiological function. The results can enhance our understanding of the roles of gut microbiota in dietary niche expansion from invertebrates to vertebrates.

Seasonal changes in diet can drive a change in the diversity of gut microbes of animals. For example, changes in the gut microbial diversity were consistent with the seasonal changes in the diets of the greater horseshoe bat (Rhinolophus ferrumequinum) (31) and the giant panda (18). However, here no significant differences in gut microbial alpha diversity were found between the insectivorous and avivorous diets, which may be due to the following two reasons. On the one hand, there is the homogeneous composition of tissues in both insects and birds despite the differences in size and nutritional quality (32). On the other hand, previous studies have shown that insect diversity in the environment decreases in autumn (26). However, our unpublished data indicated that individual dietary diversity of *I. io* did not increase in autumn, because bats prey on one bird that may be the body size and nutritional equivalent of many insects. Additionally, some avivorous individuals of *I. io* consumed not only birds but also some insects, which did not preclude the fact that insects still contributed to diet for the avivorous group. This was in line with the result that observed some gut microbes (208 ASVs) shared between insectivorous and avivorous diets. In these cases, it is reasonable that differences in gut microbial alpha diversity were not observed. Although the diversity in gut communities of *I. io* did not differ in any of the metrics we employed (alpha diversity), there was a significant difference in the gut microbial community structure (beta diversity) between diets. This finding suggested that *I. io* likely does not need to change the gut microbial diversity to adapt to the avian diet; instead, the dietary change simply requires a shift in the composition of the gut microbial community. The changes may be beneficial, because *I. io* also preys on insects in addition to birds in autumn for dietary supplementation. The results were consistent with a previous study on the American pika (Ochotona princeps), suggesting that changes in beta diversity rather than alpha diversity were detected to adapt to the novel diet of moss (33). Thus, the patterns may be common for adaptation to a novel diet in wild animals. However, prior studies showed that the bat microbiome can reflect its geographical location (34), which would include diet but also the cave the bats inhabit, water sources, and so on. Future research should collect the microbiome of environmental sites to make this association.

Previous studies have shown that the gut microbiota of bats is dominated by the bacterial phylum *Proteobacteria* and is the most compositionally distinct, followed by *Firmicutes*, *Bacteroidetes*, and *Actinobacteria* (34–37). However, in this study, *Firmicutes* (53%) and *Proteobacteria* (33%) were the two dominant phyla in the gut microbiota of *I. io*. In other mammals such as the lion (Panthera leo) (15, 38) and the baleen whales (39), as well as humans (40), *Firmicutes* and *Bacteroidetes* dominate. Therefore, our results indicated that the unique composition of the gut microbiota of *I. io* may be due to the avian diet. Such a case may be present in other avivorous bats, or even in specific carnivorous bats, but further research is needed.

We found significant differences in the relative abundance of some major bacterial phyla and genera by comparison of the compositions of gut microbes in *I. io* under different diets. At the phylum level, the relative abundance of *Firmicutes* was significantly higher under an avivorous diet than under an insectivorous diet. *Firmicutes* can produce a large amount of energy-rich short-chain fatty acids that are associated with digestion efficiency, which may be crucial for animals that need to maximize energy harvesting from their diet (41). Migratory birds had relatively higher nutritional and/or energetic value than invertebrate insects (25). Thus, one can hypothesize that an increase in *Firmicutes* may satisfy the nutrient absorption and energy extraction needs of avivorous individual bats. *Firmicutes* are also responsible for some key metabolic conversions and play an important role in polysaccharide degradation for producing energy in the human intestinal community (42). Furthermore, the change in the *Firmicutes*-to-*Bacteroidetes* ratio was associated with fat deposition and the potential for obesity, and this evidence comes from model systems and humans (41, 43–45). For example, relative to lean mice, obese mice had a significantly greater ratio of *Firmicutes* to *Bacteroidetes* (43). In humans, obese people (e.g., western European children) tend to have a higher *Firmicutes*-to-*Bacteroidetes* ratio than lean people (e.g., rural African children) (44, 45). In our study, the mean *Firmicutes*-to-*Bacteroidetes* ratio in avivorous individuals was more than two times that in insectivorous individuals. Therefore, the high *Firmicutes*-to-*Bacteroidetes* ratio in autumn in *I. io* may promote more efficient storage and/or extraction of energy from the avian diet, eventually leading to greater increases in total body fat before the bats enter into hibernation and migration.

At the genus and ASV levels, bacteria with significant changes almost all belonged to the phyla *Firmicutes* and *Proteobacteria*, which may indicate adaptation to different magnitudes of dietary nutrient ingredients and energy from insects and birds. For example, most Pseudomonas bacteria can secrete extracellular lipase, which can decompose and utilize the fat in the substrate to produce fatty acids, glycerol, aldehydes, ketones, and other substances (46). The relative abundance of Pseudomonas was significantly decreased in avivorous individuals compared to insectivorous individuals. This result may also be related to the fat storage of *I. io* before entering hibernation and migration, because with a decrease in Pseudomonas, less lipase is produced, less fat is broken down, and thus more fat is deposited. Additionally, because *Clostridium_sensu_stricto_1* and Escherichia-*Shigella* play an important role in amino acid utilization of protein diets in animals (47, 48), here significant increases in the two bacterial genera in individual bats with avian diets may function in the utilization of protein. The protein quality of birds is higher than that of insects due to the larger amount of amino acids (49, 50). As expected, body mass in individual bats with the avian diet was greater than in those with the insect diet, while forearm length was not. These results further confirmed energy harvest and nutritional utilization inferred from changes in major bacterial phyla and genera of gut microbiota in *I. io*. Thus, our results suggest that shifts in gut microbes of *I. io* can facilitate adaptation to energy and nutritional requirements for changes in foraging behavior and life history traits (i.e., hibernation and migration).

Shifting from insects to birds in the diet of *I. io* not only changed gut microbial composition but also affected microbial function. We found that bats preying on birds enriched functional categories associated with carbohydrate and nucleotide metabolism. This may be related to higher and more rapid energy expenditure for hunting birds in flight, because bats may need to fly farther, higher, and faster to prey on birds with high nocturnal migration speed (28, 51). It is therefore possible that the greater energy provided by increased carbohydrate and nucleotide metabolism met the energy requirement for hunting birds. These results suggested that the gut microbiome associated with specific metabolism-related functions plays an essential role during bats preying on birds, as well as energy harvest. However, PICRUSt2 is a predictive tool for analysis of microbial community function, with two main limitations—that rare environment-specific functions are less likely to be identified and that these functions cannot provide resolution to distinguish strain-specific functionality (52). Thus, shotgun metagenomics sequencing would be needed to reveal the functional changes related to gut microbial taxa in response to dietary shifts in further studies.

In conclusion, our results demonstrated that seasonal dietary shifts drive the marked and specific changes in the composition and function of gut microbiomes, facilitating adaptation to the absorption and utilization of energy and nutrition induced by a unique avian diet in bats. To our knowledge, this is the first study to investigate the relationships between gut microbiomes and the diets that have evolved from invertebrate insects (the ancestral diet) to small vertebrates such as birds. These results suggested that changes in gut microbiota induced by dietary shifts or possibly caused by the different microbiota transiently brought in with the consumed food allow wild animals to expand their dietary niches from invertebrate to vertebrate food resources, thereby reducing interspecific competition and enhancing fitness. Our results also provide novel insight into the role of gut bacteria in generating ecological diversity and flexibility in wild mammals. A limitation of this study is that microbial community functions in connection with the avian diets were predicted by PICRUSt2. Further manipulative experiments with shotgun metagenomics sequencing are needed to untangle the complicated host-microbiota-physiolog*y* axis, with the ultimate aim of detecting causal relationships. Further research needs to conduct physiological experiments through captive colonies of bats to assess metabolic differences among individuals fed on different diets (insects versus bird meat) to better link the changes in microbiomes to protein catabolism or other outcomes more explicitly than just body mass.

## MATERIALS AND METHODS

### Sample collection.

A total of 32 adult *I. io* males were collected from Feilong Cave (24°58.426′N, 104°52.687′E) in Xingyi, Guizhou Province, China, in June and July (summer) and October and November (autumn) in 2018. This cave is mainly a colony of *I. io* males. In the winter, individual bats either hibernate in this cave or migrate farther south to overwinter elsewhere (24). Our previous studies had shown that individual bats within this population mostly prey on insects in the summer (June to August) and on birds in the autumn (September to November) (24). Bats were captured using a mist net at the entrance to the cave after they returned from foraging (between 20:00 and 07:00). Adults were identified according to the degree of ossification of the metacarpal epiphyseal cartilages (53). We determined whether bats preyed on birds based on the collected feces containing avian feathers (22, 24). Sixteen insectivorous individuals in summer and 16 avivorous individuals in autumn were collected. Each individual bat was placed singly in a clean and sterilized cotton cloth bag. Fecal pellets were collected in a sterile 2-ml cryotube immediately after defecation and were stored on dry ice. After the bats had not defecated for more than 5 h, the bats were weighed using an electronic balance (±0.01 g; ProScale LC-50; Accurate Technology, Inc., Asheville, NC, USA), and their forearm length was measured with a digital caliper (±0.01 mm; Tesa-Cal IP67; Tesa Technology, Renens, Switzerland). In this case, the effect of recently consumed food on body mass was excluded. All captured bats were marked with aluminum alloy bat rings (5.2 mm; Porzana Ltd., Icklesham, United Kingdom) on their forearms for individual identification before they were released back into the cave. Any repeat-sampled individuals were excluded from fecal collection. The samples were transferred to the laboratory in dry ice and then stored at −80°C until DNA extraction.

### DNA extraction, PCR amplification, and sequencing.

Total DNA from collected fecal samples was extracted using an E.Z.N.A Mag-Bind soil DNA kit (Omega, Norcross, GA, USA) following the manufacturer’s instructions. The samples from summer (insectivorous) and autumn (avivorous) were divided into two batches for extraction. Negative controls were included during DNA extraction and PCR amplification to ensure that there was no contamination. The V3-V4 hypervariable region of the bacterial 16S rRNA gene was amplified using primers 341F (CCTACGGGNGGCWGCAG) and 805R (GACTACHVGGGTATCTAATCC) (54). Sample-specific 6-bp barcodes were attached to the primers for multiplex sequencing. PCRs were performed following the protocol described previously (55). After amplification, PCR products were visualized by electrophoresis using 2.0% agarose gels. PCR amplicons were purified with Agencourt AMPure XP beads (Beckman Coulter, Indianapolis, IN, USA) and quantified using the Qubit 3.0 DNA detection kit (Life Technologies, Carlsbad, CA, USA). Amplicons were pooled in equal molar ratios and paired-end sequenced (2 × 300 bp) on the Illumina MiSeq platform according to the standard protocols from Sangon Biotech Co., Ltd. (Shanghai, China).

### Sequence data processing.

Microbial raw sequences were merged by FLASH, version 1.2.7 (56), and processed using QIIME2, version 2020.2 (57). We used the DADA2 plugin in QIIME2 to denoise and quality filter reads. This step filtered out noise, removed chimeras and singletons, and finally dereplicated sequences, resulting in a series of high-resolution ASVs and a feature table of ASV counts for subsequent analysis. Taxonomy was assigned to the ASV feature table using the Native Bayes classifier in QIIME2 (58) trained against the SILVA reference database, version 138 (59) (available for download from https://www.arb-silva.de/no_cache/download/archive/release_138/Exports/). Nonbacterial ASVs and sequences identified as chloroplasts and mitochondria were excluded from the data set. To remove the influences of variable sequencing depth, we rarefied the ASV feature table to 20,000 sequences per sample according to the produced rarefaction curves (see Fig. S2 in the supplemental material) in QIIME2 for downstream analyses.

10.1128/mSphere.00467-21.2 FIG S2 Rarefaction curves for number of observed ASVs of gut microbial communities in each sample. Download FIG S2, TIF file, 1,812 kb. Copyright © 2021 Gong et al. 2021 Gong et al. https://creativecommons.org/licenses/by/4.0/ This content is distributed under the terms of the Creative Commons Attribution 4.0 International license.

### Data analysis. (i) Alpha and beta diversity analyses.

We calculated four alpha diversity metrics (observed ASVs, Shannon diversity index, evenness, and Faith’s phylogenetic diversity) for each sample using QIIME2. Differences in each measure of alpha diversity between insectivorous and avivorous diets were compared using Mann-Whitney *U* tests. Additionally, we performed simple linear regressions to test whether body mass affected microbial diversity (i.e., four alpha diversity metrics). To analyze the structure of the gut microbial community between diets, we performed principal-coordinate analysis (PCoA) using three distance matrices (Bray-Curtis, unweighted UniFrac, and weighted UniFrac distances) of beta diversity calculated in QIIME2. Tests for differences in beta diversity were performed using permutational multivariate analysis of variance (PERMANOVA) based on 999 permutations using the vegan package in R 3.5.0 (60). Additionally, we used permutational analysis of multivariate dispersions (PERMDISP) with 999 permutations to test homogeneity of dispersions for three distances using the betadisper function in the vegan package in R 3.5.0 (61).

### (ii) Microbial composition and differences analyses.

On the basis of the results of the taxonomic analysis, changes in the relative abundances of taxa in the gut microbial community between diets at the phylum and genus levels were visualized in QIIME2. Mann-Whitney *U* tests were used to compare differences in relative abundances of different taxonomic levels between insectivorous and avivorous diets. Additionally, we used a Venn diagram to show the shared ASVs across the two dietary groups. We also used linear discriminant analysis (LDA) effect size (LEfSe) with a threshold logarithmic LDA score of 2.0 to identify significantly different (*P* < 0.05) representative ASVs of gut microbial features between diets (62). We calculated the *Firmicutes*-to-*Bacteroidetes* ratio (mean relative abundance of *Firmicutes*/mean relative abundance of *Bacteroidetes* in the same dietary group) involved in host energy harvesting in order to evaluate efficient capacity for energy assimilation of gut microbiota in different diets (41).

### (iii) Predicting changes in microbial function through PICRUSt2.

To infer changes in microbial function associated with the dietary shifts, functional metagenomic prediction analysis by means of PICRUSt2 (Phylogenetic Investigation of Communities by Reconstruction of Unobserved States) (52) was performed on the ASVs within the QIIME2 environment. Predicted metagenome data were obtained using a rarefied ASV feature table (20,000 sequences per sample). Mann-Whitney *U* tests were used to compare the relative abundance of metabolic functional categories using the second-level KEGG pathways between insectivorous and avivorous diets.

### Ethics statement.

This study conformed to the ASAB/ABS Guidelines for the Treatment of Animals in Behavioral Research. All experimental procedures carried out in this study were approved by the Laboratory Animal Welfare and Ethics Committee of Northeast Normal University, China (approval number: NENU-W-2017-101). All samples were obtained without harming the study animal. After the experiments, all bats were released in good health at the cave where they were captured.

### Data availability.

Raw sequence data have been submitted to the National Center for Biotechnology Information (NCBI) Sequence Read Archive under accession number SRR12807089.
