# Supplementary material for: Seasonal Dietary Shifts Alter the Gut Microbiota of Avivorous Bats: Implication for Adaptation to Energy Harvest and Nutritional Utilization
Source: mSphere. 2021 Aug 4;6(4):e00467-21. doi: 10.1128/mSphere.00467-21 (PMC8386476; doi:10.1128/mSphere.00467-21)
Supplement: TABLE S1 [file msphere.00467-21-st001.docx]

**TABLE S1** Information of sampling, number of sequences per sample, and alpha diversity index of the gut microbiome of great evening bat in this study.

| **Sample** | **Body mass** | **Forearm length** | **Diet category** | **Sequences number** | **Observed ASVs** | **Shannon diversity** **index** | **Evenness** | **Faith’s phylogenetic diversity** |
| --- | --- | --- | --- | --- | --- | --- | --- | --- |
| S1 | 48.24 | 76.89 | insectivorous | 25451 | 296 | 4.27 | 0.75 | 48.15 |
| S2 | 48.43 | 77.84 | insectivorous | 20316 | 122 | 2.81 | 0.58 | 25.65 |
| S3 | 48.74 | 77.42 | insectivorous | 25612 | 174 | 2.01 | 0.39 | 31.31 |
| S4 | 50.61 | 73.03 | insectivorous | 21186 | 210 | 4.80 | 0.90 | 36.21 |
| S5 | 47.70 | 73.08 | insectivorous | 30144 | 271 | 4.35 | 0.78 | 46.79 |
| S6 | 49.33 | 75.49 | insectivorous | 29800 | 86 | 2.83 | 0.64 | 15.07 |
| S7 | 56.44 | 80.51 | insectivorous | 31529 | 96 | 3.10 | 0.68 | 19.60 |
| S8 | 53.20 | 77.06 | insectivorous | 24702 | 111 | 2.53 | 0.54 | 24.31 |
| S9 | 47.30 | 74.48 | insectivorous | 24053 | 59 | 1.73 | 0.42 | 12.81 |
| S10 | 48.52 | 74.17 | insectivorous | 30368 | 63 | 1.92 | 0.46 | 13.15 |
| S11 | 52.82 | 75.69 | Insectivorous | 29604 | 219 | 3.85 | 0.72 | 31.58 |
| S12 | 51.85 | 76.47 | insectivorous | 31790 | 118 | 2.87 | 0.60 | 20.56 |
| S13 | 45.52 | 74.84 | insectivorous | 27130 | 87 | 2.31 | 0.52 | 15.89 |
| S14 | 52.47 | 77.88 | insectivorous | 28713 | 362 | 4.88 | 0.83 | 37.17 |
| S15 | 51.71 | 77.76 | insectivorous | 32658 | 269 | 3.96 | 0.71 | 28.68 |
| S16 | 48.10 | 75.84 | insectivorous | 32055 | 224 | 3.42 | 0.63 | 28.45 |
| A1 | 57.98 | 75.28 | avivorous | 55341 | 265 | 2.69 | 0.48 | 48.63 |
| A2 | 60.77 | 74.75 | avivorous | 25236 | 296 | 4.19 | 0.74 | 48.49 |
| A3 | 53.82 | 72.53 | avivorous | 30525 | 13 | 1.62 | 0.63 | 2.08 |
| A4 | 63.45 | 74.03 | avivorous | 33472 | 562 | 5.39 | 0.85 | 77.05 |
| A5 | 61.77 | 76.14 | avivorous | 38038 | 169 | 3.40 | 0.66 | 31.76 |
| A6 | 57.46 | 74.01 | avivorous | 30780 | 32 | 2.21 | 0.64 | 10.30 |
| A7 | 60.07 | 72.91 | avivorous | 29554 | 15 | 1.71 | 0.63 | 2.45 |
| A8 | 56.11 | 76.66 | avivorous | 25425 | 44 | 1.62 | 0.43 | 8.73 |
| A9 | 51.75 | 73.91 | avivorous | 31553 | 25 | 1.19 | 0.37 | 6.22 |
| A10 | 63.39 | 74.54 | avivorous | 24415 | 213 | 4.10 | 0.76 | 35.76 |
| A11 | 62.00 | 74.29 | avivorous | 25132 | 95 | 1.73 | 0.38 | 21.76 |
| A12 | 64.59 | 80.17 | avivorous | 21832 | 181 | 3.38 | 0.65 | 35.22 |
| A13 | 67.13 | 77.08 | avivorous | 31240 | 21 | 0.93 | 0.31 | 5.10 |
| A14 | 56.78 | 75.36 | avivorous | 25102 | 218 | 3.59 | 0.67 | 38.79 |
| A15 | 65.17 | 75.84 | avivorous | 32639 | 17 | 1.05 | 0.37 | 2.77 |
| A16 | 57.12 | 76.01 | avivorous | 24279 | 300 | 3.98 | 0.70 | 49.38 |
