# Supplementary figures and images for: Seasonal Dietary Shifts Alter the Gut Microbiota of Avivorous Bats: Implication for Adaptation to Energy Harvest and Nutritional Utilization
Source: mSphere. 2021 Aug 4;6(4):e00467-21. doi: 10.1128/mSphere.00467-21 (PMC8386476; doi:10.1128/mSphere.00467-21)

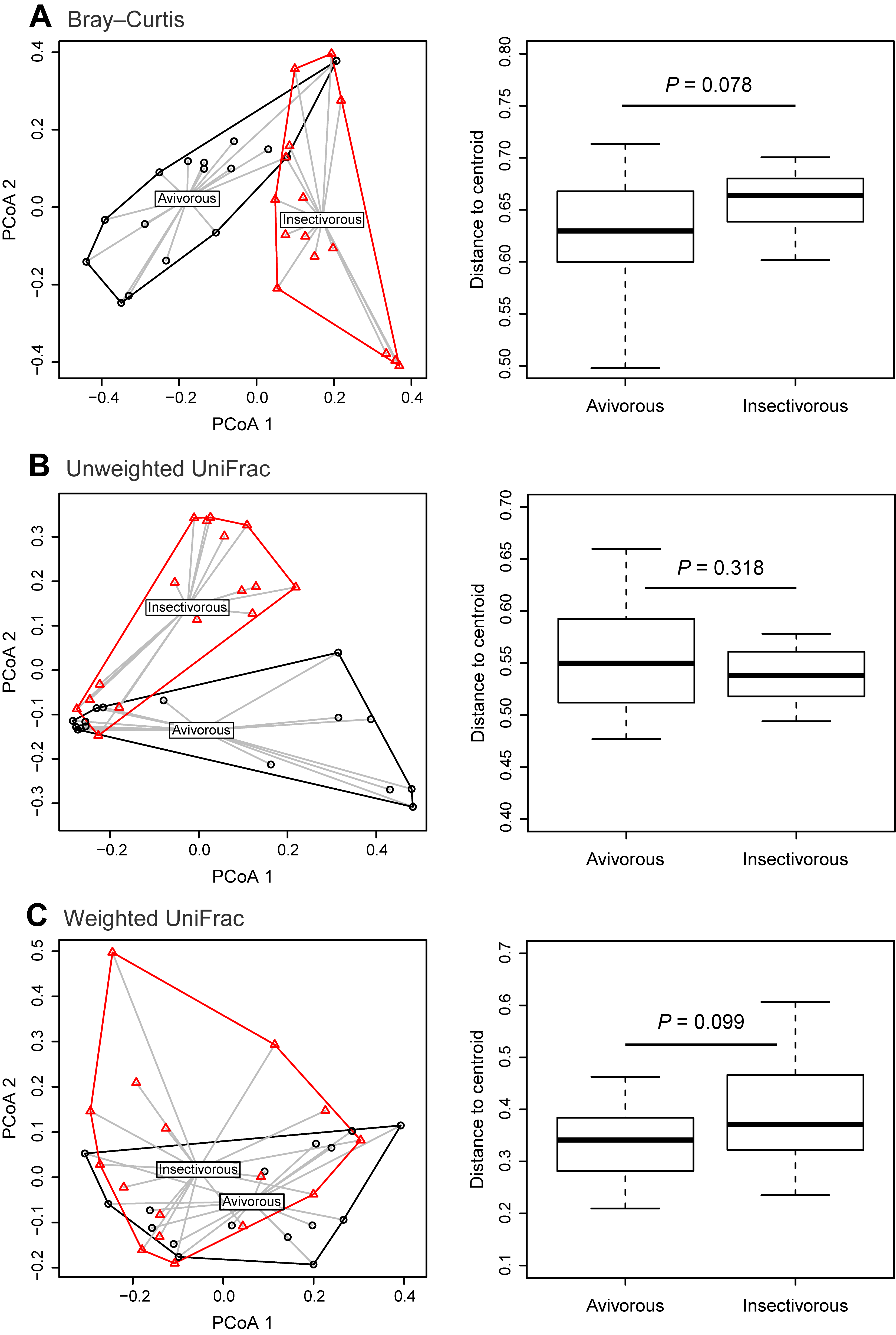

Supplement: FIG S1 [file msphere.00467-21-sf001.tif]

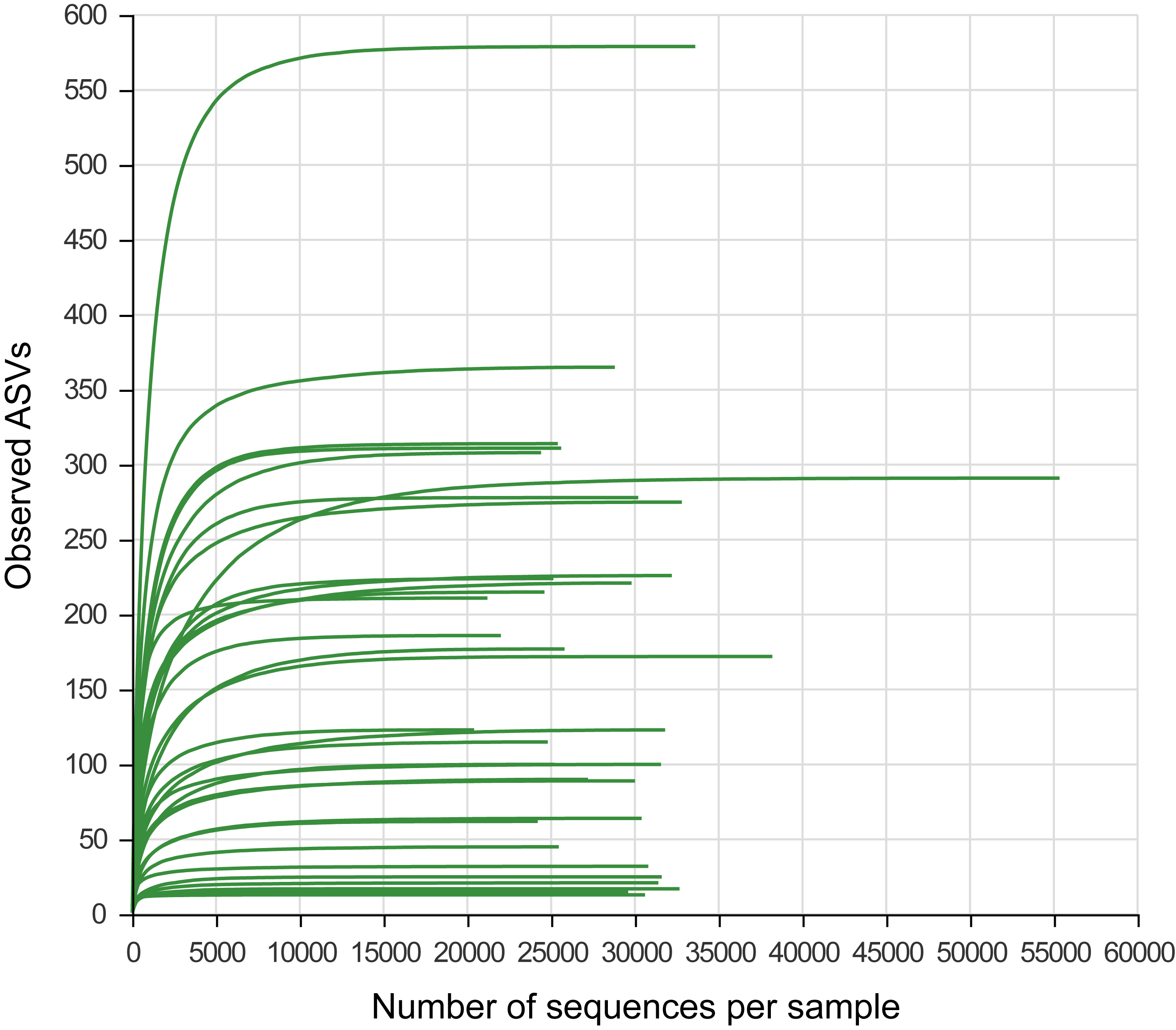

Supplement: FIG S2 [file msphere.00467-21-sf002.tif]
