# Supplementary material for: Seasonal Dietary Shifts Alter the Gut Microbiota of Avivorous Bats: Implication for Adaptation to Energy Harvest and Nutritional Utilization
Source: mSphere. 2021 Aug 4;6(4):e00467-21. doi: 10.1128/mSphere.00467-21 (PMC8386476; doi:10.1128/mSphere.00467-21)
Supplement: TABLE S2 [file msphere.00467-21-st002.docx]

**TABLE S2** Relative abundances of the six most abundant bacterial phyla and genera of the gut microbial community in great evening bats between insectivorous and avivorous diets. Values shown are means ± SE. Significant results are in bold (*P* < 0.05).

| **Taxonomic classification** | **Insectivorous (%)** | **Avivorous (%)** | ***Z*** | ***P-value*** |
| --- | --- | --- | --- | --- |
| Phylum |  |  |  |  |
| Firmicutes | 44.11 ± 5.55 | 62.38 ± 6.31 | –2.073 | **0.038** |
| Proteobacteria | 38.30 ± 5.31 | 28.25 ± 4.25 | –1.281 | 0.200 |
| Bacteroidetes | 3.97 ± 1.58 | 2.53 ± 0.84 | –0.737 | 0.461 |
| Desulfobacterota | 3.28 ± 1.25 | 0.17 ± 0.07 | –2.573 | **0.010** |
| Actinobacteria | 2.66 ± 1.15 | 0.72 ± 0.25 | –1.894 | 0.058 |
| Rs-K70_termite_group | 2.31 ± 1.06 | 0 | –2.656 | **0.008** |
| Genus |  |  |  |  |
| *Pseudomonas* | 17.55 ± 4.65 | 2.51 ± 0.66 | –3.618 | **< 0.001** |
| *Enterococcus* | 5.94 ± 1.88 | 10.61 ± 5.11 | –0.547 | 0.585 |
| *Candidatus_Arthromitus* | 12.78 ± 5.25 | 3.33 ± 3.18 | –2.681 | **0.007** |
| *Clostridium_sensu_stricto_1* | 0.64 ± 0.27 | 15.06 ± 6.25 | –3.183 | **0.001** |
| *Paeniclostridium* | 1.39 ± 1.00 | 13.88 ± 5.85 | –2.478 | **0.013** |
| *Escherichia-Shigella* | 0.06 ± 0.03 | 11.27 ± 3.01 | –4.685 | **< 0.001** |
