# Supplementary material for: Seasonal Dietary Shifts Alter the Gut Microbiota of Avivorous Bats: Implication for Adaptation to Energy Harvest and Nutritional Utilization
Source: mSphere. 2021 Aug 4;6(4):e00467-21. doi: 10.1128/mSphere.00467-21 (PMC8386476; doi:10.1128/mSphere.00467-21)
Supplement: TABLE S3 [file msphere.00467-21-st003.docx]

**TABLE S3** Significant differences in relative abundances at phylum and genus levels of the gut microbial community in great evening bats between insectivorous and avivorous diets. Values shown are means ± SD. Significance levels are *P* < 0.05.

| **Taxonomic classification** | **Insectivorous** **(%)** | **Avivorous (%)** | ***Test-statistic*** | ***P-value*** |
| --- | --- | --- | --- | --- |
| Phylum |  |  |  |  |
| Firmicutes | 44.11 ± 22.2 | 62.38 ± 25.24 | 183 | 0.03997 |
| Desulfobacterota | 3.282 ± 4.99 | 0.1747 ± 0.2652 | 60.5 | 0.01065 |
| Rs-K70_termite_group | 2.308 ± 4.246 | 0 | 80 | 0.008578 |
| Campylobacterota | 0.1194 ± 0.1343 | 2.012 ± 3.705 | 193.5 | 0.01355 |
| unclassified_d_Bacteria | 0.1347 ± 0.2151 | 0.03219 ± 0.05376 | 71 | 0.02743 |
| Genus |  |  |  |  |
| *Pseudomonas* | 17.55 ± 18.62 | 2.506 ± 2.631 | 32 | 0.0003187 |
| *Candidatus_Arthromitus* | 12.78 ± 21.01 | 3.334 ± 12.71 | 63.5 | 0.007818 |
| *Clostridium_sensu_stricto_1* | 0.6428 ± 1.068 | 15.06 ± 25.04 | 212 | 0.001558 |
| *Paeniclostridium* | 1.393 ± 4.002 | 13.88 ± 23.41 | 189.5 | 0.01399 |
| *Escherichia-Shigella* | 0.06188 ± 0.1331 | 11.27 ± 12.03 | 249 | 3.07E-06 |
| *Lactococcus* | 5.414 ± 7.182 | 1.563 ± 5.199 | 61 | 0.009477 |
| *Desulfovibrio* | 2.308 ± 4.141 | 0.06156 ± 0.1172 | 58.5 | 0.006602 |
| *norank_f_Ruminococcaceae* | 2.299 ± 3.821 | 0.01125 ± 0.02527 | 54.5 | 0.002412 |
| *norank_p_Rs-K70_termite_group* | 2.308 ± 4.246 | 0 | 80 | 0.008578 |
| *Helicobacter* | 0.1138 ± 0.1309 | 2.006 ± 3.707 | 195.5 | 0.01092 |
| *Ignatzschineria* | 2.081 ± 7.43 | 0 | 88 | 0.01851 |
| *unclassified_f_Rhizobiaceae* | 1.491 ± 3.34 | 0.4497 ± 1.546 | 55.5 | 0.004588 |
| *Sphingopyxis* | 1.567 ± 2.672 | 0 | 40 | 9.88E-05 |
| *Cetobacterium* | 0.006562 ± 0.02625 | 1.301 ± 4.632 | 177.5 | 0.01518 |
| *Luteimonas* | 0.6991 ± 1.098 | 0.1569 ± 0.2228 | 72.5 | 0.03322 |
| *Alistipes* | 0.8016 ± 1.874 | 0.005312 ± 0.02125 | 79.5 | 0.01736 |
| *norank_f_Synergistaceae* | 0.8025 ± 2.639 | 0 | 88 | 0.01851 |
| *Porphyrobacter* | 0.3191 ± 0.707 | 0.4466 ± 0.4847 | 187 | 0.01994 |
| *Dysgonomonas* | 0.6738 ± 1.742 | 0 | 80 | 0.008578 |
| *Candidatus_Soleaferrea* | 0.4888 ± 0.8099 | 0 | 72 | 0.003839 |
| *norank_f_Muribaculaceae* | 0.08344 ± 0.1998 | 0.2791 ± 0.6178 | 176 | 0.04411 |
| *norank_o_Desulfobacterales* | 0.3512 ± 0.7626 | 0 | 88 | 0.01851 |
| *unclassified_f_Intrasporangiaceae* | 0.2862 ± 1.019 | 0 | 88 | 0.01851 |
| *ZOR0006* | 0.2034 ± 0.4238 | 0.00625 ± 0.025 | 70 | 0.00628 |
| *Microbacterium* | 0.1978 ± 0.4906 | 0 | 96 | 0.03885 |
| *Gordonia* | 0.1903 ± 0.4796 | 0 | 88 | 0.01851 |
| *Blautia* | 0.02406 ± 0.09625 | 0.1656 ± 0.2878 | 175.5 | 0.01986 |
| *unclassified_d__Bacteria* | 0.1347 ± 0.2151 | 0.03219 ± 0.05376 | 71 | 0.02743 |
| *Herminiimonas* | 0.1569 ± 0.2199 | 0.002812 ± 0.01125 | 52 | 0.000783 |
| *unclassified_o_Micrococcales* | 0.1525 ± 0.5247 | 0 | 96 | 0.03885 |
| *Aliihoeflea* | 0.1413 ± 0.2512 | 0 | 80 | 0.008578 |
| *Tsukamurella* | 0.1359 ± 0.3281 | 0 | 88 | 0.01851 |
| *Breznakia* | 0.1025 ± 0.113 | 0 | 48 | 0.0002665 |
| *Gallicola* | 0.1016 ± 0.2467 | 0 | 96 | 0.03885 |
| *Vespertiliibacter* | 0 | 0.08969 ± 0.205 | 184 | 0.003839 |
| *norank_f_Burkholderiaceae* | 0.08594 ± 0.1577 | 0 | 80 | 0.008578 |
| *norank_f_Dysgonomonadaceae* | 0.075 ± 0.1743 | 0 | 96 | 0.03885 |
| *unclassified_f_Chitinophagaceae* | 0 | 0.06844 ± 0.1891 | 160 | 0.03885 |
| *Candidatus_Tammella* | 0.06688 ± 0.1447 | 0 | 96 | 0.03885 |
| *Raoultibacter* | 0.06438 ± 0.1226 | 0 | 72 | 0.003839 |
| *norank_o_RsaHf231* | 0.05906 ± 0.1254 | 0 | 80 | 0.008578 |
| *Erysipelothrix* | 0.05406 ± 0.1118 | 0 | 80 | 0.008578 |
| *unclassified_f_Xanthomonadaceae* | 0.04188 ± 0.0865 | 0.01062 ± 0.03637 | 81 | 0.03287 |
| *norank_o_Veillonellales-*  *Selenomonadales* | 0.05094 ± 0.1447 | 0 | 88 | 0.01851 |
| *norank_f_Acidaminococcaceae* | 0.05094 ± 0.1006 | 0 | 88 | 0.01851 |
| *Shewanella* | 0 | 0.04875 ± 0.09931 | 176 | 0.008578 |
| *Prevotella* | 0.005 ± 0.02 | 0.04375 ± 0.1033 | 167 | 0.0449 |
| *Crossiella* | 0.04625 ± 0.0966 | 0 | 96 | 0.03885 |
| *Erythrobacter* | 0.04094 ± 0.08496 | 0 | 88 | 0.01851 |
| *Rothia* | 0 | 0.03281 ± 0.06804 | 160 | 0.03885 |
| *Halomonas* | 0 | 0.02906 ± 0.06427 | 168 | 0.01851 |
| *Undibacterium* | 0.02906 ± 0.05914 | 0 | 96 | 0.03885 |
| *Xanthobacter* | 0 | 0.01562 ± 0.02683 | 168 | 0.01849 |
| *norank_f_Methyloligellaceae* | 0.01 ± 0.02236 | 0 | 96 | 0.0388 |
| *norank_f_Pedosphaeraceae* | 0 | 0.00875 ± 0.02053 | 160 | 0.03885 |
| *norank_o_Rhizobiales* | 0.008125 ± 0.01965 | 0 | 96 | 0.03885 |
