# Supplementary material for: Seasonal Dietary Shifts Alter the Gut Microbiota of Avivorous Bats: Implication for Adaptation to Energy Harvest and Nutritional Utilization
Source: mSphere. 2021 Aug 4;6(4):e00467-21. doi: 10.1128/mSphere.00467-21 (PMC8386476; doi:10.1128/mSphere.00467-21)
Supplement: TABLE S4 [file msphere.00467-21-st004.docx]

**TABLE S4** PICRUSt2 showing predicted relative abundance of metabolic-related functional categories based on the second level KEGG pathways. Values shown are means ± SE. Significant results are in bold (*P* < 0.05).

| **KEGG Level 2_ Metabolic Pathway** | **Insectivorous (%)** | **Avivorous (%)** | ***Z*** | ***P-value*** |
| --- | --- | --- | --- | --- |
| Carbohydrate metabolism | 13.76 ± 0.22 | 14.80 ± 0.31 | –2.337 | **0.019** |
| Global and overview maps | 11.52 ± 0.16 | 10.70 ± 0.17 | –2.714 | **0.007** |
| Amino acid metabolism | 10.47 ± 0.19 | 9.56 ± 0.20 | –2.751 | **0.006** |
| Energy metabolism | 6.14 ± 0.09 | 5.96 ± 0.07 | –1.508 | 0.132 |
| Metabolism of cofactors and vitamins | 5.64 ± 0.09 | 5.76 ± 0.09 | –0.754 | 0.451 |
| Nucleotide metabolism | 4.99 ± 0.12 | 5.77 ± 0.15 | –3.053 | **0.002** |
| Lipid metabolism | 3.38 ± 0.06 | 3.48 ± 0.05 | –1.131 | 0.258 |
| Xenobiotics biodegradation and metabolism | 2.56 ± 0.19 | 2.05 ± 0.10 | –2.035 | **0.042** |
| Metabolism of other amino acids | 2.20 ± 0.06 | 2.24 ± 0.02 | –0.754 | 0.451 |
| Glycan biosynthesis and metabolism | 1.69 ± 0.09 | 1.71 ± 0.03 | –0.905 | 0.366 |
| Biosynthesis of other secondary metabolites | 1.59 ± 0.03 | 1.44 ± 0.04 | –2.261 | **0.024** |
| Metabolism of terpenoids and polyketides | 1.52 ± 0.03 | 1.35 ± 0.04 | –2.563 | **0.010** |
